# The gut microbe pair of *Oribacterium* sp. GMB0313 and *Ruminococcus* sp. GMB0270 confers complete protection against SARS-CoV-2 infection by activating CD8+ T cell-mediated immunity

**DOI:** 10.1080/19490976.2024.2342497

**Published:** 2024-04-18

**Authors:** Mingda Wang, Enkhchimeg Lkhagva, Sura Kim, Chongkai Zhai, Md Minarul Islam, Hyeon J. Kim, Seong-Tshool Hong

**Affiliations:** a Department of Biomedical Sciences, Jeonbuk National University Medical School, Jeollabuk-Do, South Korea; b Department of Critical Care Medicine, Shandong Provincial Hospital affiliated with Shandong First Medical University, Jinan, China; c College of Food and Drugs, Luoyang Polytechnic, Animal Diseases and Public Health Engineering Research Center of Henan Province, Luoyang, Henan Province, China; d BioLabs-LA at the Lundquist Institute for Bio Medical Innovation at Harbor UCLA, SNJ Pharma Inc, Torrance, CA, USA

**Keywords:** SARS-CoV-2, COVID-19, gut microbiome, host-directed vaccine, CD8+ T cell, universal vaccine, betacoronavirus

## Abstract

Despite the potential protective role of the gut microbiome against COVID-19, specific microbes conferring resistance to COVID-19 have not yet been identified. In this work, we aimed to identify and validate gut microbes at the species level that provide protection against SARS-CoV-2 infection. To identify gut microbes conferring protection against COVID-19, we conducted a fecal microbiota transplantation (FMT) from an individual with no history of COVID-19 infection or immunization into a lethal COVID-19 hamster model. FMT from this COVID-19-resistant donor resulted in significant phenotypic changes related to COVID-19 sensitivity in the hamsters. Metagenomic analysis revealed distinct differences in the gut microbiome composition among the hamster groups, leading to the identification of two previously unknown bacterial species: *Oribacterium* sp. GMB0313 and *Ruminococcus* sp. GMB0270, both associated with COVID-19 resistance. Subsequently, we conducted a proof-of-concept confirmation animal experiment adhering to Koch’s postulates. Oral administration of this gut microbe pair, *Oribacterium* sp. GMB0313 and *Ruminococcus* sp. GMB0270, to the hamsters provided complete protection against SARS-CoV-2 infection through the activation of CD8+ T cell mediated immunity. The prophylactic efficacy of the gut microbe pair against SARS-CoV-2 infection was comparable to, or even superior to, current mRNA vaccines. This strong prophylactic efficacy suggests that the gut microbe pair could be developed as a host-directed universal vaccine for all betacoronaviruses, including potential future emerging viruses.

## Introduction

The COVID-19 pandemic has recently emerged as a new infectious disease, presenting a significant threat to global public health. Understanding the origin of SARS-CoV-2,^1–3^ the etiological agent for COVID-19, is paramount in addressing this global crisis.^4,5^ Genome analyzes have revealed similarities between the genomes of several zoonotic betacoronaviruses found in bats and pangolins and that of SARS-CoV-2, suggesting a possible zoonotic origin. However, none of these viruses closely match SARS-CoV-2, leaving questions about its emergence unanswered.^6,7^ Recently, a novel approach involving whole prototype proteome sequence analysis has provided insights into the origin of SARS-CoV-2. This analysis suggests that the virus emerged through an unusual replacement event, where a motif in the spike protein of SARS-CoV-1 was replaced by a motif from a membrane protein of the malaria parasite *Plasmodium malaria*.^8^

Since its emergence, SARS-CoV-2 has spread rapidly worldwide, leading to the continuous emergence of new variants such as Omicron and its subsequent variants.^9^ This ongoing evolution suggests that completely eradicating the virus from our society is now unlikely. Epidemiological and virological evidence indicates that SARS-CoV-2 has become entrenched in human society, resembling the seasonal behavior of the influenza virus.^10^ As a result, it is anticipated that COVID-19 will periodically resurge, similar to influenza outbreaks, necessitating stringent measures to control the ispread of the disease.^11^

Betacoronaviruses are increasingly emerging and spreading rapidly in modern society due to factors such as heightened human-wildlife contact, global interconnectedness, climate change, and the international trade of animals and their products.^12^ Over the past two decades alone, three betacoronavirus species – SARS, MERS, and COVID-19—have emerged. ^13–15^ This trend suggests a likelihood of further emergence of deadly betacoronavirus species in the near future. Considering the potential for seasonal COVID-19 outbreaks, there is a pressing need for the development of a universal prophylactic vaccine against betacoronaviruses. Despite the critical necessity for such a vaccine, this poses a significant scientific challenge. Since each betacoronavirus species has distinct antigens, it would not be possible to develop a single antigen capable of broadly targeting all betacoronavirus antigens for a universal vaccine.^16^

While developing a betacoronavirus universal vaccine with an antigen broadly covering all betacoronaviruses is not feasible, a host-directed vaccine approach could offer a promising alternative. Recent studies have highlighted the critical role of the gut microbiome, consisting of microbes in the human gut, in various immune functions. While developing a betacoronavirus universal vaccine with an antigen broadly covering all betacoronaviruses is not feasible, a host-directed vaccine approach could offer a promising alternative. Through the microbiome-immune axis, the gut microbiome contributes to cytokine production, immune system regulation, T cell production, and overall immune system balance, providing protection against various infectious diseases.^17,18^ This suggests that specific gut microbes could serve as candidates for a betacoronavirus universal vaccine. In our research, we identified a pair of gut microbes that conferred complete protection against SARS-CoV-2 infection. This finding, supported by metagenomic analysis of the gut microbiome and a proof-of-concept animal experiment adhering to Koch’s postulates, underscores the significance of the gut microbiome in modulating host immunity and might position it as an attractive candidate for the development of a universal betacoronavirus vaccine.

## Results

### Fecal microbiota transplantation from a COVID-19-resistant human into a lethal COVID-19 animal model induced phenotypic changes associated with COVID-19 sensitivity in the animals

One unique aspect of SARS-CoV-2 is that the incidence rate of COVID-19 does not seem to correlate with the vaccination rate.^19^ For instance, in the Republic of Korea, where 87.1% of the population has been vaccinated, predominantly with mRNA vaccines, there is a notably high incidence rate of COVID-19 per capita. This incidence rate is at least 10 times higher than that of most third-world countries, where the vaccination rate ranges from 5% to 30% (source: https://ourworldindata.org). This discrepancy between vaccination rates and the COVID-19 incidence strongly suggests the existence of a hidden critical factor in protecting against SARS-CoV-2 infection.

Considering the significant role of the gut microbiome in human immunity to viral pathogens,^17,18^ we suspected that the hidden factor protecting against SARS-CoV-2 infection could be a specific gut microbe within the gut microbiome. To identify such a microbe, we conducted fecal microbiota transplantation (FMT) experiments. Specifically, we transplanted stool from an individual who had never contracted COVID-19 and had no history of immunization into a lethal COVID-19 animal model, the *Phodopus roborovskii* SH101 strain. This particular hamster strain is highly susceptible to SARS-CoV-2 infection,^20,21^ making it an ideal model for studying COVID-19 pathogenesis. To prepare the animals for FMT, we depleted their gut microbiomes using a combination of antibiotics and antifungal agents. Subsequently, we administered the stool sample orally to the hamsters and then infected them with SARS-CoV-2 via the nasal route.

**Figure 1.**
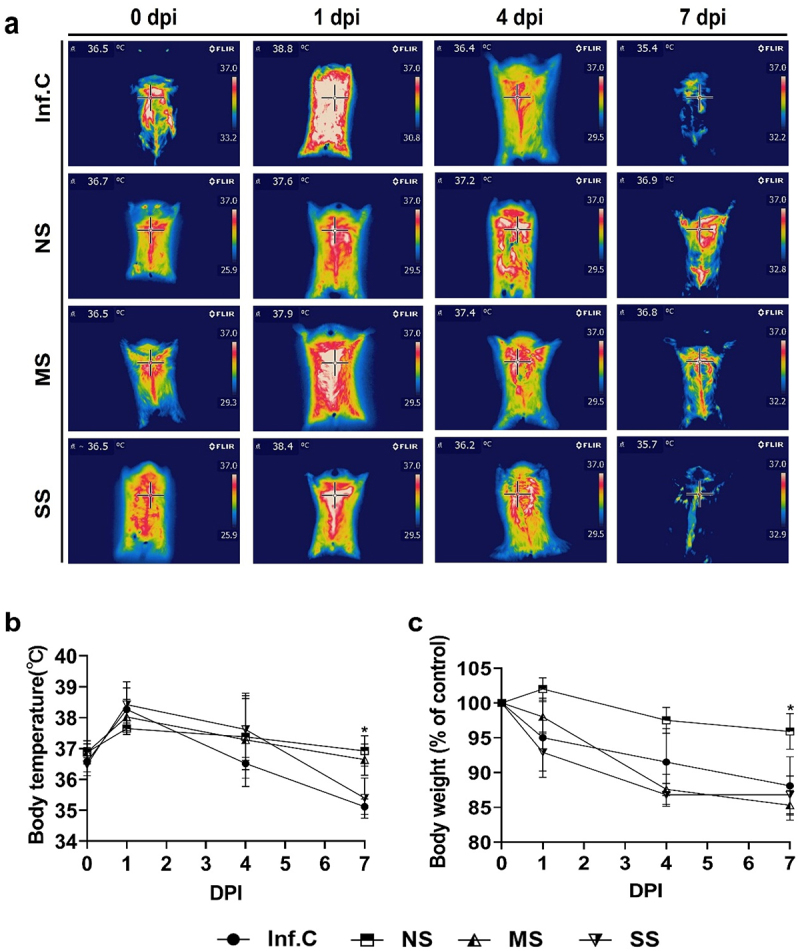
Grouping of the SH101 Roborovski hamsters based on the severity of infection symptoms following fecal transplantation of feces from a COVID-19 resistant human. The gut microbiomes of 6-week-old SH101 Roborovski hamsters were depleted by daily administration of antibiotic/antifungal mixtures. Subsequently, a fresh fecal sample was collected from an individual with no history of COVID-19 or vaccination and was orally administered to the hamsters (*n* = 20). One week later, the SH101 hamsters were intranasally infected with a lethal amount of SARS-CoV-2 virus, 50 μL of 10^5^ TCID50. Body temperature and body weight were monitored and recorded daily for 7 days post-infection (dpi). Excluding the 5 hamsters that died upon infection, the remaining 15 hamsters were grouped by the severity of their symptoms; 6 hamsters in the severe symptom (SS) group, 5 hamsters in the mild symptom (MS) group, and 4 hamsters in the no symptom (NS) group, compared to the infection control (Inf.C, *n* = 6) group. (a) Thermal imaging of the body after the infection of SH101 hamsters. (b) The body temperature of the indicated groups of SH101 hamsters for 7 dpi (mean ± SD). (c) The body weight (% of control) in the indicated groups of SH101 hamsters for 7 dpi (mean ± SD). Significance was statistically analyzed and marked on the graphs as *p < 0.05.

Interestingly, the hamsters that received FMT before SARS-CoV-2 infection exhibited immediate differences in response compared to the infection control group. Out of the 20 hamsters that underwent FMT, five were found dead even after overnight housing while the remaining 15 hamsters displayed varying responses to SARS-CoV-2 infection. It should be noted that the five deceased hamsters were excluded from the gut microbiome analysis due to damage to the abdominal area caused by neighboring surviving hamsters, which prevented the isolation of intact intestinal matter for analysis. The surviving 15 hamsters were monitored for their body temperature and were categorized into three groups on 7 days post-infection (dpi): the no symptom (NS) group (*n* = 4, average body temperature of 36.9 ℃), the mild symptom (MS) group (*n* = 5, average body temperature of 36.6 ℃), and the severe symptom (SS) group (*n* = 6, average body temperature of 35.4 ℃), compared to the infection control group (*n* = 5, average body temperature of 35.1 ℃) (Figure 1). Body weight data showed non-significant differences in the SS but significant difference in the NS group compared to the infection control group, supporting the result of body temperature data. The hamsters in the SS group were observed to have diarrhea, probably due to disturbed gut microbiota (Supplemental Figure S1). It should be noted that despite the nasal inoculation of SARS-CoV-2, which typically leads to the universal development of COVID-19 in SH101 Roborovski hamsters, the observation of COVID-19-resistant hamsters (NS group) is noteworthy.

### Diagnostic examinations revealed that the COVID-19 sensitivity of the SH101 Roborovski hamsters had been altered following FMT

**Figure 2.**
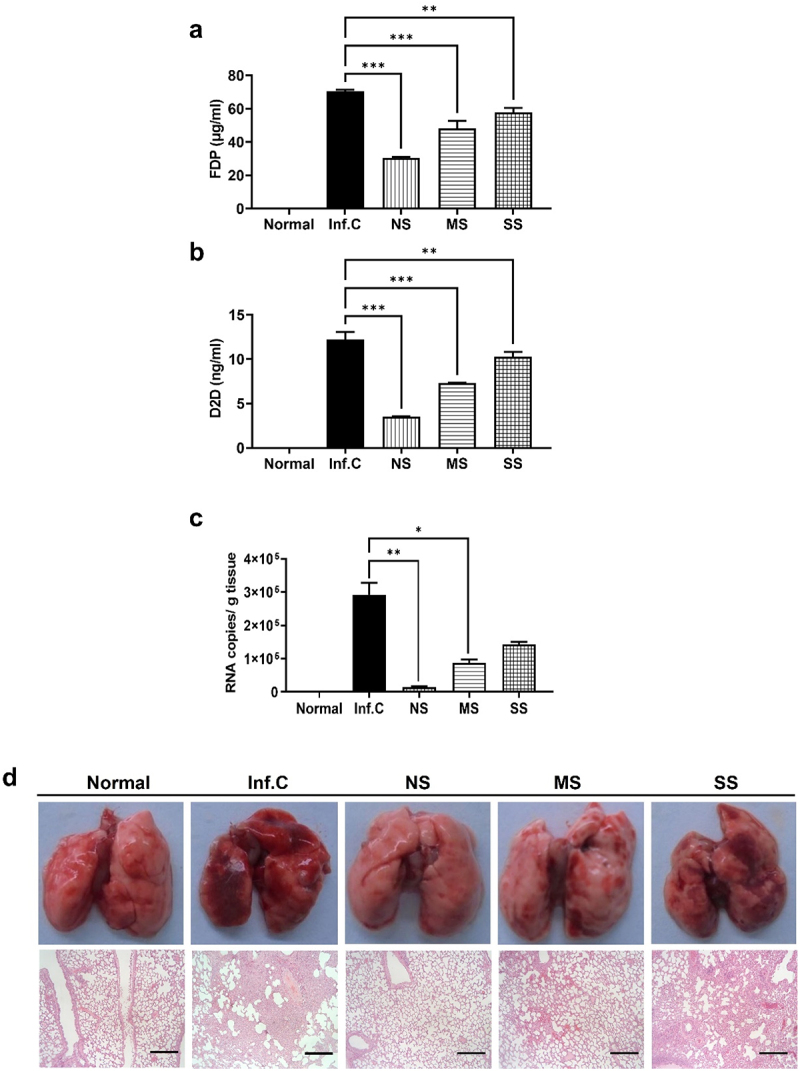
Identification of COVID-19 resistant animals after fecal transplantation of feces from a COVID-19 resistant human. The groups with varying degrees of infection symptoms (NS, MS, and SS) were assessed for systemic inflammation and viral titer in comparison to both the no-infection control (Normal) and infection control (Inf.C) groups. (a-b) Quantitative analysis of diagnostic tests for inflammation in the blood of the indicated group by cytokine ELISA test against (a) FDP and (b) D2D (mean ± SD). (c) The SARS-CoV-2 RNA copy numbers per gram of lung tissue in the indicated groups using RT-qPCR (mean ± SD). (d) The representative images of the lungs (top) and tissue sections (bottom) after H&E staining. Scale bar, 100 μm. Significance was statistically analyzed and marked on the graphs as *p <0 .05, **p < 0.01, and ***p <0 .001, respectively. NS, MS, and SS represent the No Symptom, Mild Symptom, and Severe Symptom groups, respectively. FDP and D2D represent the fibrin degradation products and D-dimer, respectively.

The clinical observations of the hamsters post-FMT prompted us to conduct diagnostic tests on the animals. We initially assessed the blood concentrations of fibrin degradation products (FDP) and D-dimer (D2D), which serve as indicators of a systemic inflammation. As shown in Figure 2a,b, the serum levels of FDP and D2D in the NS group were slightly higher than those in the healthy control group but remained significantly lower than those in the mock control group. The serum levels of FDP and D2D in the MS group fell between those of the NS and mock control group. It should be noted that the blood levels of FDP and D2D were higher than those in the healthy control group, despite the absence of clinical symptoms of SARS-CoV-2 infection in the NS group as shown in Figure 1. This suggests the presence of mild inflammation in the NS group, despite the hamsters being clinically healthy.

Consistent with the clinical observations of the hamsters, viral counting by RT-PCR revealed minimal detection of the SARS-CoV-2 virus in the NS group, suggesting clearance of SARS-CoV-2 (Figure 2c). SARS-CoV-2 was moderately detected in the MS group, while the quantity of SARS-CoV-2 in the SS group was higher than that in the mock control group. Overall, the quantification results of SARS-CoV-2 virus aligned well with the clinical observations of the hamsters in Figure 1. Histological examinations further confirmed that hamsters in the NS group were not affected by COVID-19, while those in the MS group exhibited moderate symptoms and those in the SS group showed severe symptoms (Figure 2d). As expected, the H&E staining of lung sections revealed heavy inflammation in the lungs of the mock control and SS groups, moderate inflammation in the MS group, and little to no inflammation in the NS group and healthy control group (Figure 2d).

These diagnostic examinations are well matched with the clinical observations presented in Figure 1, confirming that the symptoms observed in the hamsters were indeed due to SARS-CoV-2 infection. In conclusion, the altered sensitivity of SH101 Roborovski hamsters to SARS-CoV-2 following FMT underscores the significant role of the gut microbiome in determining susceptibility to COVID-19. Furthermore, it suggests the presence of intestinal microbes that could potentially confer complete protection against COVID-19 to their host.

### The gut microbiomes of the hamsters, which exhibited varying degrees of sensitivity to SARS-CoV-2 following FMT, displayed significant differences from one another

Since the fecal microbiome may not fully represent the entire gut microbiome,^22^ the entire gastrointestinal (GI) content of each hamster from the SS, MS, and NS groups was collected and used for genomic DNA extraction for gut microbiome analysis. This analysis targeted the V3 and V4 hypervariable regions of the 16S rRNA gene. A total of 2,070 operational taxonomic units (OTUs) were identified, representing 22 phyla, 153 families, and 278 genera (Supplemental Figure S2). The predominant phyla in the gut microbiome were Bacteroidetes and Firmicutes, which is consistent with the typical composition of a mammalian gut microbiome (Supplemental Figure S2a). As expected, the classified OTUs at lower taxonomic levels revealed compositional differences among the three hamster groups (Supplemental Figure S2b).

**Figure 3.**
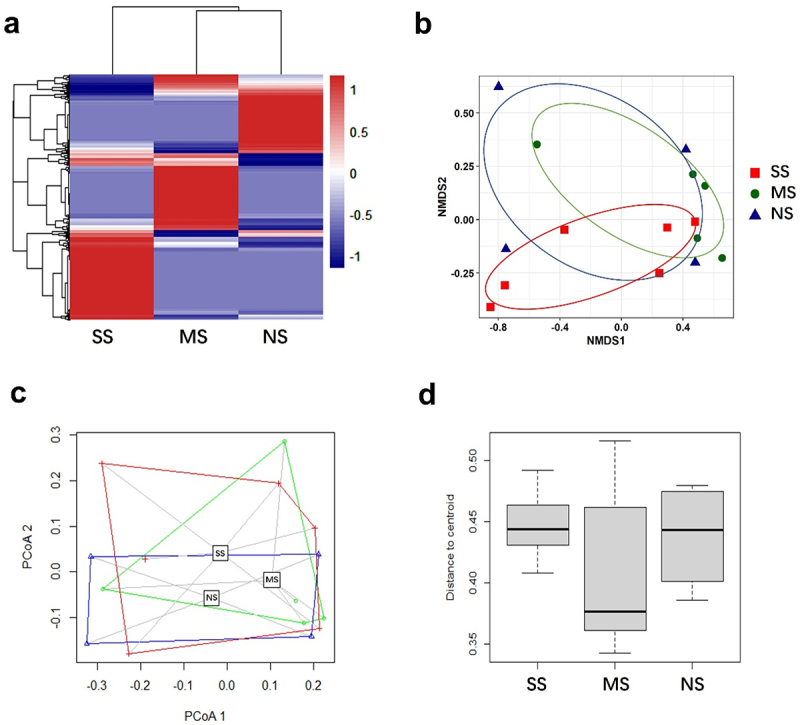
The differences in gut microbiota composition and abundance of operational taxonomic units (OTUs) among COVID-19 infected hamster groups exhibiting varying degrees of sensitivity to SARS-CoV-2 infection. The entire gastrointestinal (GI) content from hamsters in each group was used to prepare total genomic DNA. It should be noted that 16S sequencing of gut microbiota was conducted only on the animal groups that underwent fecal transplantation, excluding the normal group and the infection control group that did not undergo fecal transplantation. Gut microbiome analysis was conducted on the isolated genomic DNAs, targeting the V3 and V4 hypervariable regions of the 16S rRNA gene, revealing 2,070 operational taxonomic units (OTUs). (a) Heatmap of the microbial composition for the SS, MS, and NS groups, based on the mean value of normalized OTUs. The dendrogram represents the clustering of microbiome composition based on species-level similarity. (b) Community distribution was calculated from normalized log-transformed abundance values and presented by non-metric multidimensional scaling (NMDS) plots based on Bray-Curtis distances by using species-level OTUs. (c-d) Principal component analysis (PCoA) (c) and the distance to the group centroid (d) were calculated with 999 permutations using the vegan package. NS, MS, and SS represent the No Symptom, Mild Symptom, and Severe Symptom groups, respectively.

The measurement of α-diversity metrics, including richness (ACE and Fisher) and evenness (Shannon, Evenness, Simpson, and InvSimpson), did not indicate significant differences among the groups (Supplemental Figure S2c). The α-diversity indices suggested similar levels of diversity in the gut microbiome, despite the observed differences in composition among the SS, MS, and NS groups. However, β-diversity analyzes, such as Bray-Curtis distance, nonmetric multidimensional scaling (NMDS) ordination plot, principal coordinate analysis (PCoA), and the measurement of the distance of the centroid on the PCoA plot of diversity metrics (Figure 3), confirmed the compositional disparities in the gut microbiomes of each hamster group.

The observed compositional differences in the gut microbiome among the hamster groups led us to investigate the microbial community network. Co-occurrence network analysis of the gut microbiome revealed distinctions among the three hamster groups (Supplemental Figure S2 and Table S1). The co-occurrence network of the SS and MS groups displayed similar characteristics, while the NS group exhibited the lowest number of nodes and edges but the highest modularity (0.844). The community structure of the overall gut microbiome indicated that the diversity of the gut microbiome in the NS group was lower compared to the MS and SS groups. However, the high modularity of the NS gut microbiome community indicated that the gut microbiome in the NS group was well-organized and more distinct compared to the SS and MS groups. This finding suggests the potential presence of intestinal microbes in the NS group that may confer resistance to SARS-CoV-2 infection.

### Intestinal microbes potentially associated with either protection against or aggravation of COVID-19 were identified

**Figure 4.**
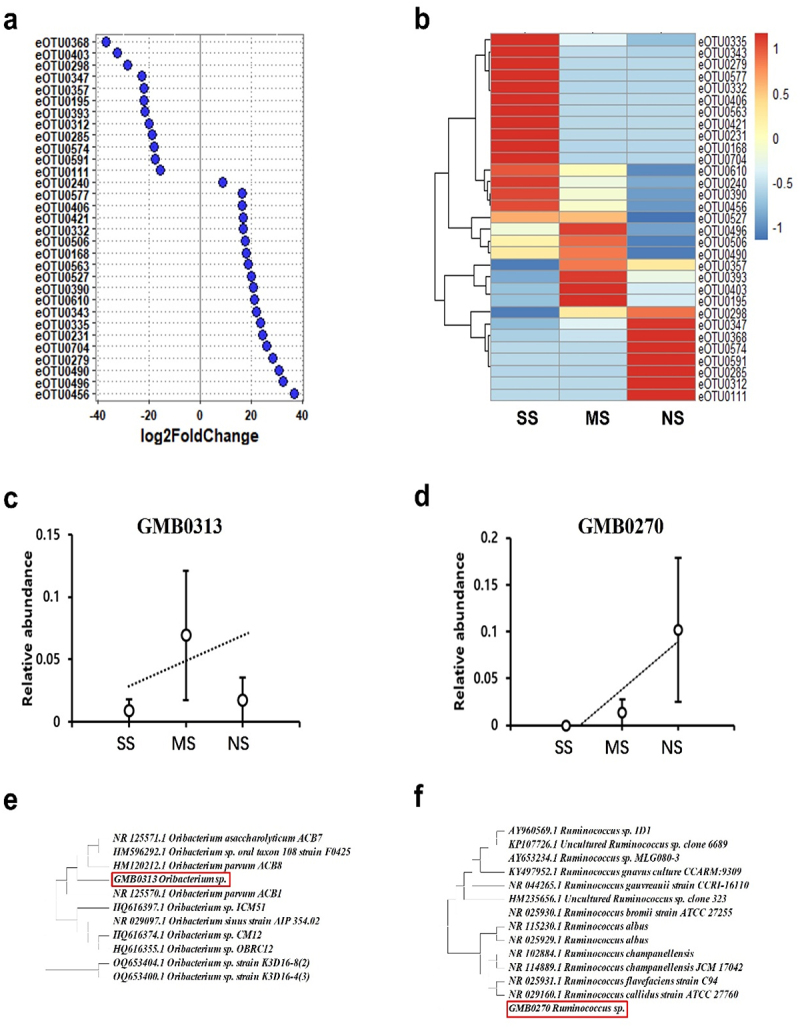
Identification of gut microbes responsible for resistance against COVID-19. (a) DESeq2 analysis was conducted to identify differentially abundant bacterial species in the NS, MS, and SS groups. The data were presented as Log2fold change for 31 eOtus, where 12 eOtus exhibited negative values in Log2fold, signifying differential abundance in the NS group, while 19 eOtus showed positive values in Log2fold, indicating differential abundance in the SS group. (b) The differential abundance of the 31 OTUs of interest was visualized more clearly in a clustered heatmap. (c-d) the relative abundance of *Oribacterium* sp. GMB0313 (c) and *Ruminococcus* sp. GMB0270 (d) in the NS, MS, and SS groups. (e-f) Phylogenetic and molecular evolutionary analyses using MEGA version 11 to determine genetic relationships with identified species. Maximum likelihood trees were constructed for known *Oribacterium* and *Oribacterium* sp. GMB0313 (e) and for known *Ruminococcus* and *Ruminococcus* sp. GMB0270 (f). Database sequence accession numbers for known organisms are provided alongside species names. NS, MS, and SS represent the No Symptom, Mild Symptom, and Severe Symptom groups, respectively.

The notable differences in the gut microbiome prompted us to investigate the identification of gut microbes that could potentially have either a protective or aggravating role in relation to COVID-19. We utilized DESeq2, a method for assessing differential abundance in microbiota composition, to identify OTUs that exhibited differential abundance in the gut microbiome of each group (Figure 4a). A total of 31 OTUs were found to have significantly different abundance (adjusted *p*-value of 0.05 or lower) between the NS and SS groups. The actual abundance of these 31 OTUs was visualized as a heatmap using unsupervised hierarchical clustering (Figure 4b). Among the 31 OTUs, 12 were found to be enriched in the NS group (Supplemental Table S2), while 19 were enriched in the SS group (Supplemental Table S3). Based on the sequences of these OTUs, microbial species were identified using the GMB Database for species-level classification. It was observed that several OTUs were associated with the same or closely related species. For instance, eOTU0403, eOTU0285, and eOTU0574 belonged to the same species (Supplemental Table S2). Since OTUs target a variable region of the 16S rRNA rather than the complete 16S rRNA region required for species identification, it is possible that the 16S rRNA sequence of a bacterial species could be fragmented into multiple OTUs.

After removing OTUs representing the same microbes, unsupervised hierarchical clustering revealed six microbes in the NS group (Supplemental Table S2) and nine microbes enriched in the SS group (Supplemental Table S3). The abundance of microbes in the SS group aligns well with previous clinical observations on the prevalence of Prevotella and Bacteroides in COVID-19 patients.^23^ In the NS group, two bacterial species, represented by eOTU0368 and eOTU0195/eOTU0393, were identified as the most promising candidates for conferring COVID-19 resistance to their host. These microbes were previously unknown species belonging to *Oribacterium* sp. and *Ruminococcus* sp., respectively (Supplemental Table S2). The presence of bacteria from the *Ruminococcus* genus, although species-level identification has not been achieved,^24,25^ has frequently been found in healthy controls, which strongly supports the validity of our findings. Linear correlation analysis further validated the association of *Oribacterium* sp. GMB0313 and *Ruminococcus* sp. GMB0270 with COVID-19 resistance (Figure 4c,d). The relative abundance of *Oribacterium* sp. GMB0313 was 0.009 ± 0.022 in SS, 0.069 ± 0.115 in MS, and 0.017 ± 0.035 in NS, respectively. Similarly, the relative abundance of *Ruminococcus* sp. GMB0270 was 0 ± 0 in SS, 0.013 ± 0.03 in MS, and 0.102 ± 0.154 in NS, respectively. These data suggest the association of these bacteria with COVID-19 resistance.

### *The animal experiments verified that the pair of* Oribacterium *sp. GMB0313 and* Ruminococcus *sp. GMB0270 provided comprehensive protection against SARS-CoV-2 infection in the host*

**Figure 5.**
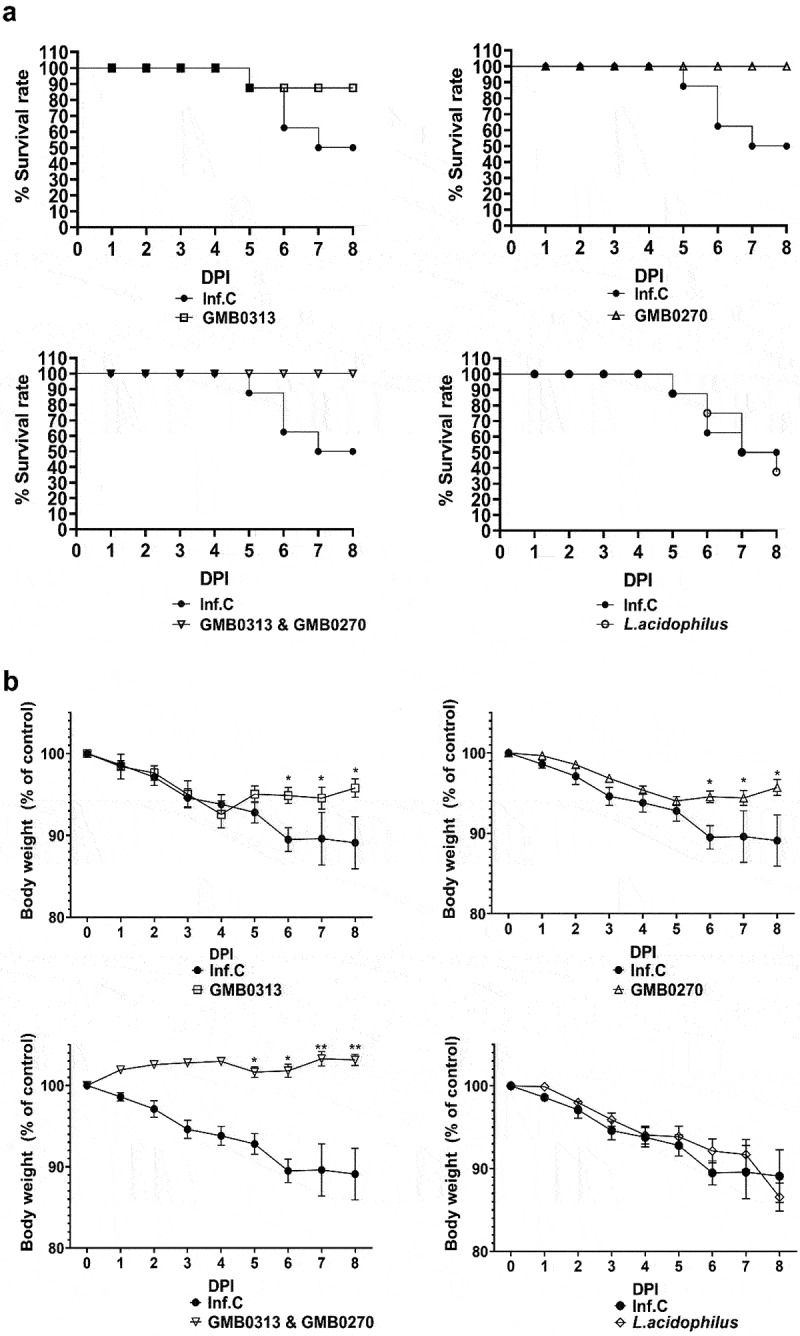
The survival rate and body weight of SARS-CoV-2-infected SH101 hamsters fed with gut microbes responsible for resistance against COVID-19. The gut microbiomes of 6-week-old SH101 Roborovski hamsters underwent depletion through daily administration of antibiotic/antifungal mixtures. Following microbiome depletion, the hamsters were randomly divided into groups receiving either PBS as infection control (Inf.C) or the cultures of *Oribacterium* sp. GMB0313, *Ruminococcus* sp. GMB0270, combination of *Oribacterium* sp. GMB0313 and *Ruminococcus* sp. GMB0270, and *Lactobacillus acidophilus* as intestine bacteria control (*n* = 8 per group). These microorganisms were orally administered to the hamsters at a dosage of 1 × 10^9^ CFU in 100 μL of PBS per day. After two weeks of daily administration, the SH101 hamsters were intranasally challenged with a lethal amount of SARS-CoV-2 virus. Daily recordings of survival rate (a) and body weight (b) were maintained for 7 days (mean ± SD). Significance was statistically analyzed and marked on the graphs as *p < 0.05 and **p < 0.01.

To establish a definitive relationship between the identified microbes and COVID-19 protection, an experiment was conducted using SARS-CoV-2 infection. The microbes, *Oribacterium* sp. GMB0313 and *Ruminococcus* sp. GMB0270, which were obtained from the Gut Microbe Bank (GMB), the largest collection of gut microbe species from humans (available at https://www.gmbank.org), were used. The infection control group consisted of SH101 Roborovski hamsters infected with SARS-CoV-2, and they exhibited typical clinical symptoms of COVID-19, such as snuffling, labored breathing, dyspnea, cough, hunched posture, progressive weight loss, ruffled fur, high fever, and shaking chills (Figure 5 and Supplemental Figure S4). Furthermore, the clinical symptoms and disease progression of the SARS-CoV-2-infected hamsters colonized with a typical probiotic strain, *Lactobacillus acidophilus*, were virtually identical to the infection group (Figure 5a,b). In contrast, the hamsters colonized with both *Oribacterium* sp. GMB0313 and *Ruminococcus* sp. GMB0270 showed no clinical symptoms of SARS-CoV-2 infection. Both bacterial groups provided protection against SARS-CoV-2 infection, as demonstrated by the survival rate (Figure 5a) and changes in body weight (Figure 5b). Notably, the combination of both species offered complete protection against COVID-19 to the host. These results definitively show that *Oribacterium* sp. GMB0313 and *Ruminococcus* sp. GMB0270 provide complete protection against SARS-CoV-2 infection in the host.

Gross examination of the lung specimens revealed a distinct uneven distribution of inflammation, with a predominant presence of pneumonia observed on the right side, notably in the infected group without treatment as well as the group treated with *L. acidophilus* (Figure 6a). This uneven pattern of pneumonia is a characteristic feature of COVID-19 associated with SARS-CoV-2 infection.^26,27,28^ In contrast to the infection control group, weak signs of pneumonia were observed in the group treated with *Oribacterium* sp. GMB0313 alone, while no signs of pneumonia were observed in the group treated with *Ruminococcus* sp. GMB0270 alone or in the group co-fed with *Oribacterium* sp. GMB0313 and *Ruminococcus* sp. GMB0270. The histological examinations confirmed these findings, showing heavy inflammation in the control group, slight inflammation in the GMB0313group, and almost no inflammation in the GMB0270 group as well as the co-feeding group (Figure 6b). It is worth mentioning that there are also huge differences in inflammation markers (Figure 6c,d) as well as the cytokine levels (Figure 6e–g) in the blood of hamsters in different groups, which is consistent with the observed symptoms. Moreover, quantitative RT-PCR analysis of viral RNA was consistent with the pathology results, with high levels of viral RNA detected in the control group, low levels in the GMB0313 group, and no detectable levels of viral RNA in the GMB0270 group as well as the co-feeding group (Figure 6h). In conclusion, complete protection against SARS-CoV-2 infection in the hamsters was achieved by feeding them the pair of *Oribacterium* sp. GMB0313 and *Ruminococcus* sp. GMB0270. This work, which demonstrates the etiological link between COVID-19 protection and the pair of *Oribacterium* sp. GMB0313 and *Ruminococcus* sp. GMB0270 based on Koch’s postulates, reports for the first time a definitive role of the microbiome in COVID-19 protection and identification of its responsible specific gut microbial species.

**Figure 6.**
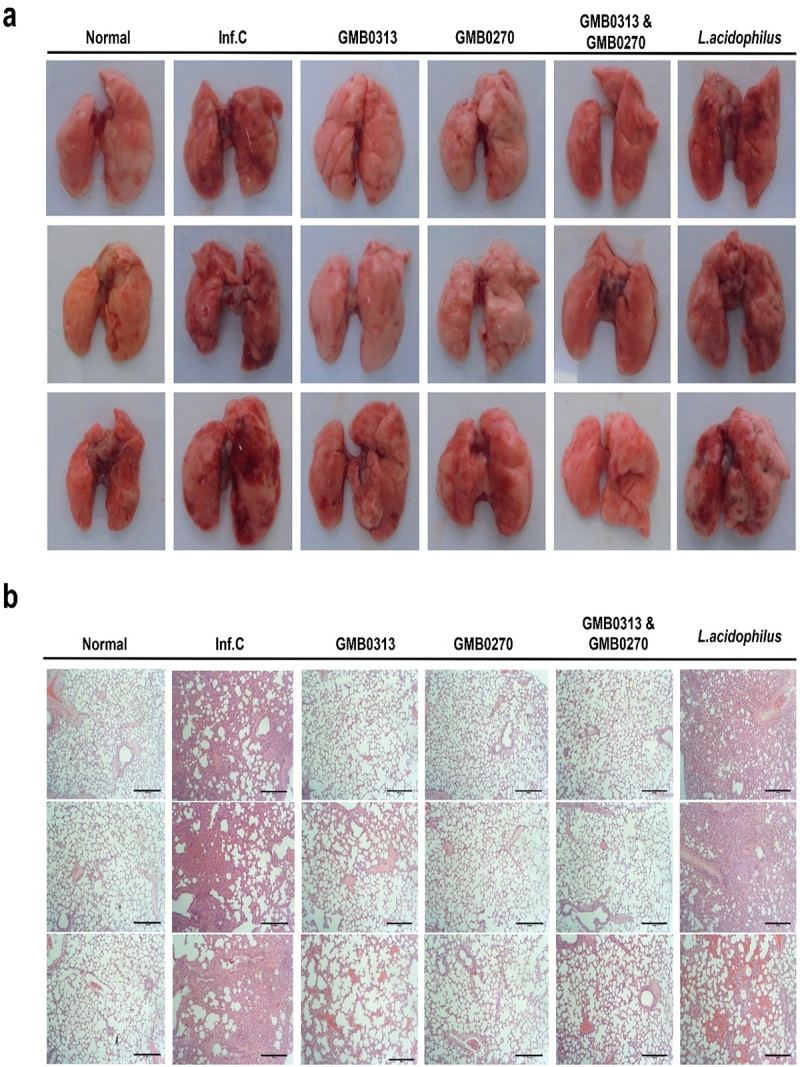
The inflammation levels and viral titers in the lungs of SARS-CoV-2 infected SH101 hamsters, which received gut microbes responsible for resistance against COVID-19. The gut microbiomes of 6-week-old SH101 Roborovski hamsters underwent depletion through daily administration of antibiotic/antifungal mixtures. Following microbiome depletion, the hamsters were randomly divided into groups receiving either PBS as infection control (Inf.C) or the cultures of *Oribacterium* sp. GMB0313, *Ruminococcus* sp. GMB0270, combination of *Oribacterium* sp. GMB0313 and *Ruminococcus* sp. GMB0270, and *L. acidophilus* as intestine bacteria control (*n* = 8 per group). These microorganisms were orally administered to the hamsters at a dosage of 1 × 10^9^ CFU in 100 μL of PBS per day. After two weeks of daily administration, the SH101 hamsters were intranasally challenged with a lethal amount of SARS-CoV-2 virus and compared with uninfected control group (Normal) and infected control (Inf.C). (a-b) All hamsters were sacrificed on 8 dpi, and fresh lung tissues were obtained for morphological observation (a) as well as hematoxylin and eosin (H&E) staining of lung sections (b). (c-g) Systemic inflammation was assessed using cytokine ELISA against (c) fibrin degradation products (FDP), (d) D-dimer (D2D), (e) IFN-γ, (f) IL-10, and (g) IL-4 (mean ± SD). (h) Quantitation of SARS-CoV-2 viral titers per gram of lung tissue in the indicated groups by RT-qPCR (mean ± SD). Significance was statistically analyzed and marked on the graphs as *p < 0.05, **p < 0.01, and ***p < 0.001.

**Figure 6.**
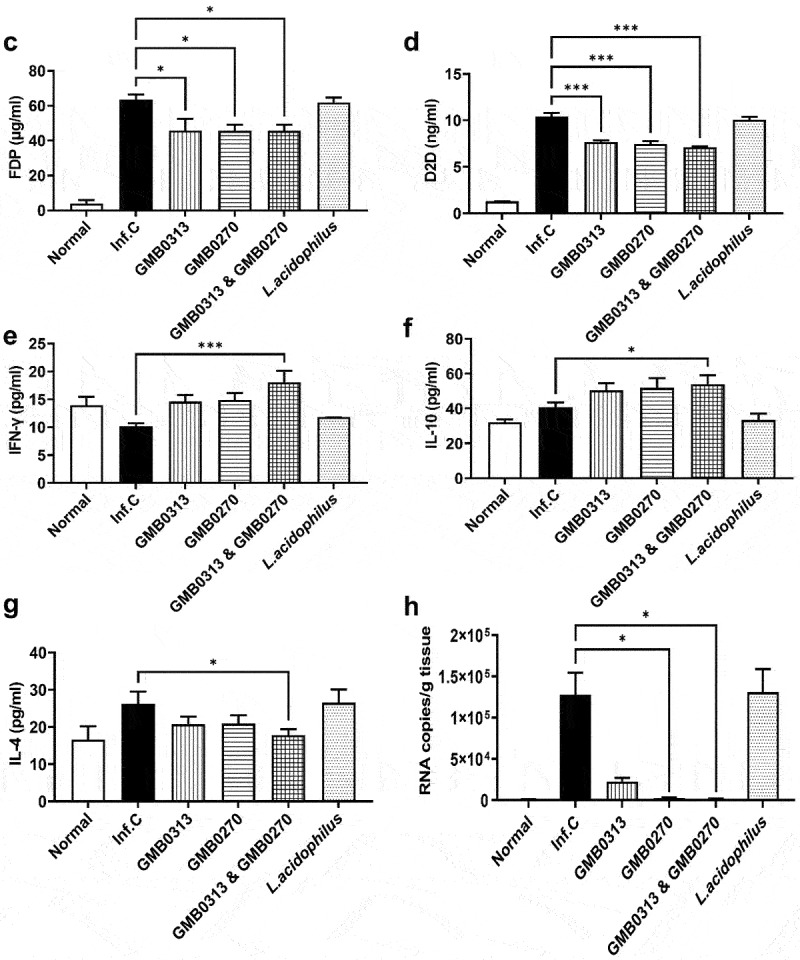
(Continued).

### *The pair of* Oribacterium *sp. GMB0313 and* Ruminococcus *sp. GMB0270 bestowed COVID-19 resistance on their host by activating CD8+ cells*

To explore the mechanism underlying the observed COVID-19 protection in the NS group, we collected blood samples from each group before and after SARS-CoV-2 infection. Immunophenotyping of whole peripheral blood was conducted using flow cytometry with CD3, CD4, CD8, CD14, and CD16 antibodies (Figure 7). Before SARS-CoV-2 infection, a significant expansion of CD8+ cells was observed compared to the mock control group in the hamsters colonized with *Oribacterium* sp. GMB0313, *Ruminococcus* sp. GMB0270 and the pair of *Oribacterium* sp. GMB0313 & *Ruminococcus* sp. GMB0270 (Figure 7a). However, there was no expansion observed in the immunological markers for helper T cells, natural killer (NK) cells,^29^ and macrophages, indicating no increase in these immune cells after colonization.^30^ Interestingly, these patterns were maintained even after SARS-CoV-2 infection (Figure 7b). The increased percentage of CD8+ cells was consistently observed in the hamsters colonized with *Oribacterium* sp. GMB0313 and *Ruminococcus* sp. GMB0270, regardless of SARS-CoV-2 infection (Figure 7a,b). In contrast, the percentages of other immune cells such as helper T cells, NK cells, and macrophages did not change after colonization with *Oribacterium* sp. GMB0313 and *Ruminococcus* sp. GMB0270 (Figure 7a,b). These immunophenotyping results clearly indicate that colonization with the pair of *Oribacterium* sp. GMB0313 and *Ruminococcus* sp. GMB0270 in the hamsters led to the expansion of cytotoxic CD8+ T cells, which provide protection against SARS-CoV-2 infection to the host.

**Figure 7.**
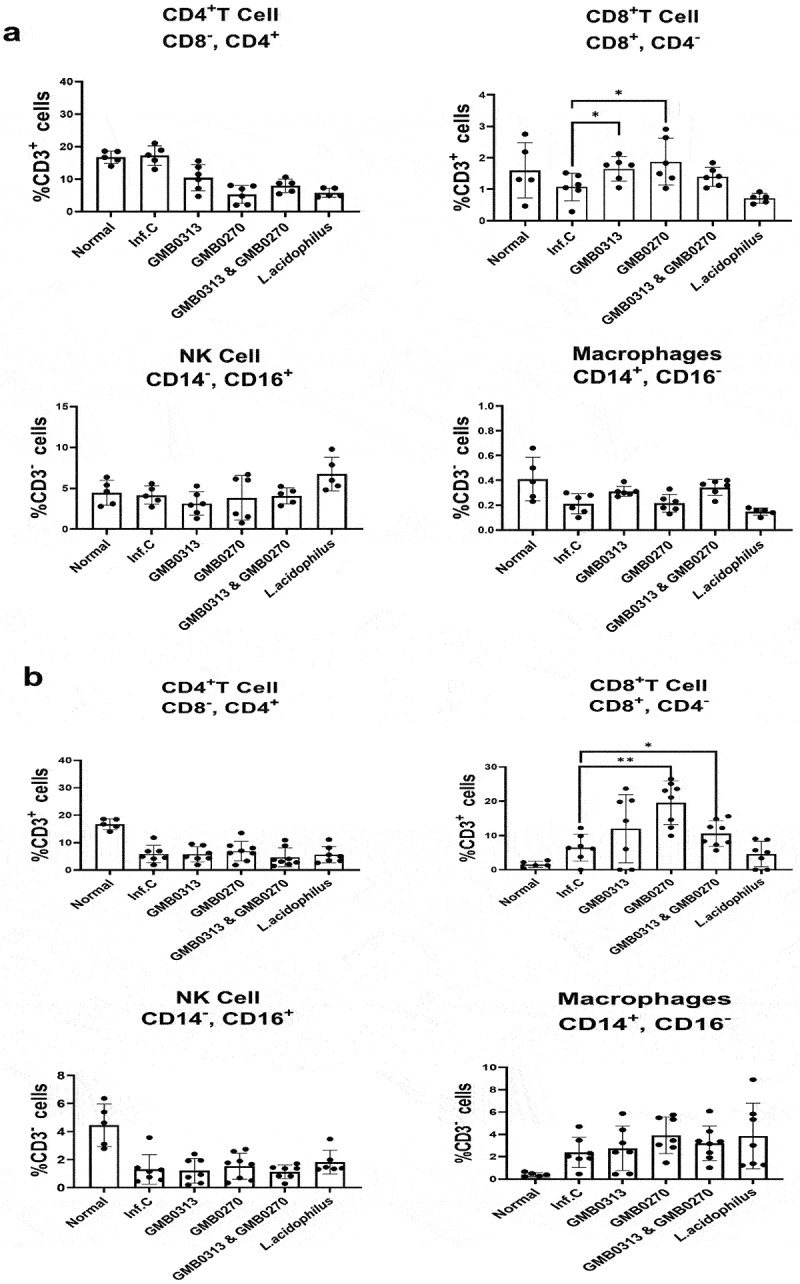
Flow cytometry analysis of immune cell populations in SARS-CoV-2 infected SH101 hamsters that were fed gut microbes responsible for resistance against COVID-19. (a-b) Immunomarkers for T helper cells, natural killer (NK) cells, and macrophages were quantified before (a) and after (b) infection with SARS-CoV-2 using the peripheral blood from SH101 hamsters fed the indicated microbes or PBS as mock control. Data on graphs represent mean ± SD. Significance was statistically analyzed and marked on the graphs as *p < 0.05 and **p < 0.01. (c) The manual gating strategy for immune cell subpopulations and visualization of unbiased clustering of single-cell multidimensional data for the immune cell panel.

**Figure 7.**
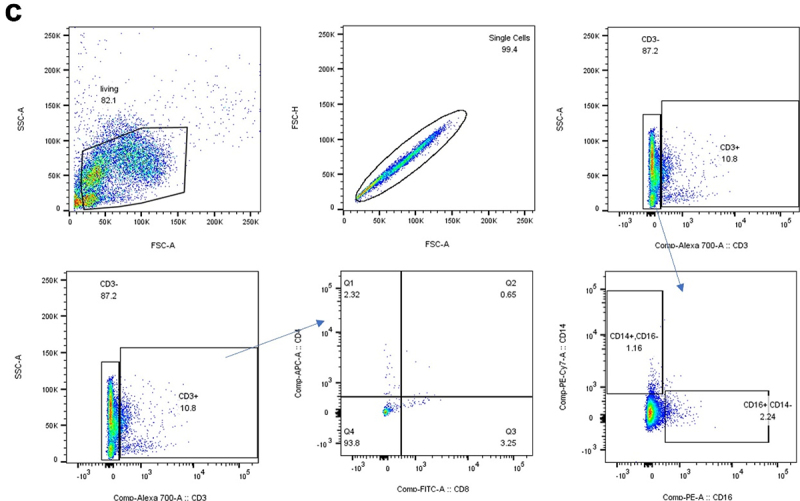
(Continued).

## Discussion

The gut microbiome plays a crucial role in the overall health and well-being of the host. Its impact on the immune system is particularly profound, as the gut microbiome has a significant influence on the development and functioning of both innate and adaptive immune responses through the microbiome-immune axis. This interaction helps provide protection against various infectious diseases by training and modulating the immune system.^31^ The importance of the gut microbiome in infection is evident from studies that have shown marked differences in bacterial diversity and composition between patients with infectious diseases, such as COVID-19 and MERS-CoV, and healthy individuals.^32,33,34^ Consistent with these findings, the gut microbiomes of the hamster groups with different degrees of SARS-CoV-2 sensitivity in this study exhibited distinct differences from each other (Figures 3,4). This observation, coupled with previous research on the compositional differences of the gut microbiome in patients and healthy individuals, suggests that the gut microbiome could be a crucial hidden factor following SARS-CoV-2 infection.

While the gut microbiome’s significance in protecting against infectious diseases, such as COVID-19, is well recognized, specific gut microbes providing this protection have never been identified at the species level. Analysis of the gut microbiome’s effect on phenotypes, including infectivity, is significantly hindered by individual genetic variation. To identify gut microbes offering protection against COVID-19, we mitigated the influence of host genes by comparing COVID-19 infectivity in genetically homogenous inbred hamsters after modifying their gut microbiome through FMT. This approach accurately pinpointed the gut microbes conferring protection against COVID-19 (Figure 4).

It is widely recognized that cytotoxic CD8+ T cells play a crucial role in combating viral infections by eliminating virus-infected cells and producing effector cytokines.^21,35^ Recent studies have highlighted the importance of CD8+ T cell-mediated immunity in COVID-19.^36^ Patients with COVID-19, especially those with severe disease, often exhibit a significant decrease in CD8+ T cells compared to healthy individuals without a history of COVID-19 infection.^35,37,38^ Conversely, healthy populations with no history of COVID-19 infection often display elevated levels of CD8+ T cells.^39^ Animal studies have also supported the role of CD8+ T cells in protection against SARS-CoV-2 infections.^40,41^ These findings align perfectly with the results of this study, emphasizing the significance of CD8+ T cells in protecting against SARS-CoV-2 infection (Figure 7).

Despite mounting evidence underscoring the importance of the gut microbiome in influencing phenotypes and diseases, much of the current research is confined to observational studies heavily reliant on metagenomic next-generation sequencing (mNGS) methods. While mNGS is a valuable tool for exploring the human gut microbiome, it tends to yield primarily phenomenological insights. To establish a causal link between specific gut microbes and diseases or phenotypes, conducting a proof-of-concept animal experiment that adheres to Koch’s postulates is essential. In this context, our study, validated through such an animal experiment adhering to Koch’s postulates, would significantly contribute to advancing our understanding of the gut microbiome’s role by providing the etiological connection between COVID-19 protection and the gut microbe pair of *Oribacterium* sp. GMB0313 and *Ruminococcus* sp. GMB0270.

Remarkably, this gut microbe pair provided complete protection against SARS-CoV-2 infection (Figures 5,6), with a comparable or even superior level of protection compared to the current mRNA COVID-19 vaccines from Pfizer and Moderna.^42,43^ Since the gut microbe pair confers protection through host-directed vaccination by activating CD8+ cells (Figure 7), rather than relying on antigen-induced active immunity, it suggests that this gut microbe pair may offer broad protection against various pathogens. The broad immunity provided by host-directed vaccination implies that this gut microbe pair could be developed as a universal vaccine capable of protecting against all betacoronaviruses, including future emerging viruses.

## Conclusions

In this study, we identified a pair of gut microbes, *Oribacterium* sp. GMB0313 and *Ruminococcus* sp. GMB0270, which plays a protective role against SARS-CoV-2 infection. Using SH101 Roborovski hamsters, a lethal animal model of COVID-19, we conducted fecal microbiota transplantation from an individual with no history of COVID-19 infection or immunization. This induced diverse phenotypic changes in COVID-19 sensitivities, including complete protection against COVID-19. Through comparative metagenome analysis of these animal groups, we identified the two novel gut microbes that confer protection against SARS-CoV-2 infection. Colonizing this microbial pair in the hamsters resulted in complete protection against SARS-CoV-2 infection by activating the CD8+ T cell population. Given that this gut microbe pair enhances host immunity to protect against SARS-CoV-2 infection, it holds potential for development as a universal vaccine for betacoronaviruses.

## Materials and methods

### Fecal microbiota transplantation (FMT)

The gut microbiomes of 6-week-old, male SH101 Roborovski hamsters (Alpha biochemicals Co., South Korea) were depleted by the daily administration of antibiotic/antifungal mixtures, azithromycin (15 mg/kg), neomycin (25 mg/kg), ciprofloxacin (20 mg/kg), and miconazole (30 mg/kg), for 2 days. Subsequently, a fresh fecal sample was collected from an individual with no history of COVID-19 and orally administered to the SH101 Roborovski hamsters to establish a de novo microbiome (*n* = 20). Unfed SH101 hamsters served as controls (*n* = 6). One week after feeding, the SH101 hamsters were infected intranasally with a lethal amount of SARS-CoV-2 virus, 50 μL of 10^5^ TCID50. Daily observations of body temperature and body weight were conducted.

### Viruses and cells

The SARS-CoV-2 strain HB-01 was from the National Culture Collection for Pathogens (NCCP) of the Korea Illness Control and Anticipation Organization (KDCA). The total genome for this SARS-CoV-2 virus has been submitted to GISAID (identifier: BetaCoV/Wuhan/IVDC-HB-01/2020| EPI_ISL_402119) and kept within the China National Microbiological Information Center (Accession number NMDC10013001 and genome promotion number MDC60013002–01). Seed stocks of SARS-CoV-2 were arranged and the infection was confined to Vero E6 cells. These cells were maintained in Dulbecco’s Modified Eagle Medium (DMEM) supplemented with 10% fetal bovine serum (FBS), 100 IU/mL penicillin, and 100 μg/mL streptomycin. The cells were then incubated at 37°C with 5% CO2.

### SARS-CoV-2 infection

The SH101 Roborovski (*Phodopus roborovskii* strain SH101) hamsters were an in-house research strain at Jinis Inc. (Wanju, Jeonbuk, Republic of Korea) and were obtained from Alpha Biochemicals Co (Jeonju, Jeonbuk, Republic of Korea). Hamsters of appropriate age were selected for experimentation and housed in separate cages with access to food (D12450B; Research Diets Inc.) and water. The animals were kept in a controlled environment in the ABL3 laboratory at Jeonbuk National University, with humidity maintained at 50% ± 5% and temperature at 20°C ±1°C, and subjected to a light-dark cycle with lights on from 8:00 pm each day. Once acclimatized to the laboratory conditions, the hamsters were intranasally inoculated with 50 μL of SARS-CoV-2 solution containing 10^5^ TCID50.

### Body temperature measurement

We assessed the body temperature of the animals using high-precision thermal imaging. Each hamster’s entire body was examined using a FLIR infrared imager, and the data were analyzed using the DirA Program (FLIR Tools).^44^ The temperature was recorded at the highest point near the lung, close to the heart, in each hamster. All data are presented as mean ± standard deviation and compared using paired Student’s t-test.

### Quantification of SARS-CoV-2

Total RNA was extracted from the supernatant of organ homogenates using the RNeasy Mini Kit (QIAGEN, Hilden, Germany) and reverse transcribed using the Primer Script RT Reagent Kit (Takara, Japan) following the manufacturer’s instructions. RT-qPCR reactions were performed using the Power Up SYBR Green Master Mix Kit (BIO-RAD, California, USA), and samples were processed using the following cycling protocol: 50°C for 2 min, 95°C for 3 min, followed by 45 cycles of 95°C for 15 s and 60°C for 30 s, then 95°C for 20 s, 55°C for 1 min, and 95°C for 50 s. The primer sequences used for RT-qPCR targeted the envelope (E) gene of SARS-CoV-2 and were as follows: forward: 5’-GCCTCTTCTCGTTCCTCATCAC-3’, reverse: 5’-AGCAGCATCACCGCCATTG-3’. PCR products were verified by sequencing on an ABI 3730 DNA sequencer (Applied Biosystems, Waltham, USA) using the dideoxy method. The obtained sequencing reads were compared with those from the NCBI database. SYBR green real-time PCR standard curves were generated by serial 10-fold dilutions of recombinant plasmids with known copy numbers ranging from 7 × 10^7^ to 7 × 10^1^ copies/μL. These dilutions are tested by plotting the nucleic acid copy number against the corresponding threshold cycle value (Ct) and used as quantification criteria to construct a standard curve. Results are expressed as log10 transformed number of genome equivalent copies per milliliter of sample. The Ct value of each sample was used to quantify the virus by using the standard curve.

### Quantitation of fibrin degradation products and D-dimer

The hypercoagulable state of the SARS-CoV-2-infected animals was analyzed with fibrin degradation products (FDP) and D-dimer (D2D) assays. Whole blood was collected in a 1.5 mL Eppendorf tube by cardiac puncture. Blood serum was separated by centrifugation at 2,500 × g for 10 min and stored at 25°C until used. The concentrations of FDP and D2D in serum samples were determined using the double antibody sandwich method with hamster FDP and hamster D2D ELISA kits (Hamster ELISA kit, MyBioSource). Quantification of FDP or D2D was conducted on micro-ELISA strip plates precoated with hamster anti-FDP or anti-D2D monoclonal antibody. The samples for quantitative analysis were prepared by diluting them 10 times with Sample diluent solution as per the kit’s manual. Subsequently, 50 μL of each diluted sample was dispensed into the wells of a micro-ELISA plate coated with antibodies against FDP or D2D. The plate was then incubated at 37°C for 60 minutes to facilitate the reaction. Following incubation, the chromogen solution was added and allowed to react for 15 minutes. The reaction was terminated by adding the stop solution. Immediately after stopping the reaction, concentrations of FDP and D2D were determined from the OD values of each sample using a standard curve.

### Hematoxylin and eosin (H&E) staining of tissue

Fresh organs were promptly isolated following the demise of the animals and preserved in 10% formalin. These organs were then encased in paraffin and sliced into microscope slides (5 μm). After eliminating the paraffin from the glass slides using 70°C water, the slides were air-dried and baked at 65°C for over 12 hours. Sections of the organs were stained with standard hematoxylin and eosin (H&E) stain. The stained histologic sections were examined using an Apero ScanScope FL (AmScope, USA).

### DNA isolation, library preparation and 16S amplicon sequencing of intestinal microbiota

Total gut microbiota genomic DNA from 15 hamsters was extracted using the phenol-chloroform isoamyl alcohol extraction protocol, as described previously.^45^ Briefly, the samples suspended in lysis buffer (200 mM NaCl, 200 mM Tris-HCl (pH 8.0), 20 mM EDTA) were subjected to bead beating for processing, and genomic DNA was extracted from the aqueous phase using phenol:chloroform:isoamyl alcohol. DNA was precipitated by adding 3 M sodium acetate followed by isopropanol. After washing with 70% ethanol and drying, the DNA pellet was dissolved in TE buffer (10 mM Tris-HCl pH 8.0, 1 mM EDTA). DNA quantification was performed using a Biospec-nano spectrophotometer (Shimadzu Biotech). It is important to note that 16S sequencing of gut microbiota was conducted only on the animal groups that underwent fecal transplantation, excluding the normal group and the infection control group that did not undergo fecal transplantation. The sequencing samples are prepared according to the Illumina 16S Metagenomic Sequencing Library protocols. The 16S rRNA genes were amplified using 16S V3-V4 primers (16S Amplicon PCR Forward Primer: 5‘TCGTCGGCAGCGTCAGATGTGTATAAGAGACAGCCTACGGGNGGCWGCAG; 16S Amplicon PCR Reverse Primer: 5’ GTCTCGTGGGCTCGGAGATGTGTATA AGAGACAGGACTACHVGGGTATCTAATCC). The genomic DNA (gDNA) samples were initially amplified with 16S V3-V4 primers, followed by a limited cycle amplification step to incorporate multiplexing indices and Illumina sequencing adapters.^46^ Subsequently, the final products were normalized and pooled using PicoGreen. The size of the libraries was confirmed using the TapeStation DNA screentape D1000 (Agilent), and sequencing (2 × 300) was performed using the MiSeq™ platform (Illumina) following the standard protocol. To enhance genome assembly, the paired-end reads obtained from Next Generation Sequencing (NGS) were merged using FLASH (Fast Length Adjustment of Short reads).^47^ The amplicon error was modeled from merged fastq using DaDa2, which filtered out noise sequence, corrected errors in marginal sequences, removed chimeric sequences, and eliminated singletons.^48^ Subsequently, the sequences were dereplicated. The Q2-Feature classifier, a Naive Bayes classifier trained based on the SILVA reference (region V3-V4) database (https://www.arb-silva.de/), was employed to classify the dataset used in the experiment.^49^ The q2-diversity was utilized for diversity calculation and statistical tests.^48^ The metagenomics data, operational taxonomic unit (OTU), and taxonomic classification tables were imported into the phyloseq (1.28.0) package in R version 3.6.1.^50^ Unclassified phyla were removed for further analysis. The OTU data was converted to metagenomeseq object and normalized by cumulative-sum-scaling (CSS), which is specifically designed for metagenome data in the bioConductor package metagenomeSeq (1.16.0).^51^

### Diversity and abundance evaluation of microbiome

Alpha diversity metrics were computed using the phyloseq package. To detect differences in richness and alpha diversity between groups, the Kruskal-Wallis rank sum test was applied. Taxa with a total relative abundance of less than 5% were collapsed into “other” at each taxonomy level before plotting. Beta diversity metrics were calculated and visualized using log-transformed, normalized OTU data in the phyloseq package, including Bray-Curtis dissimilarity.^50^ To identify statistical differences in beta diversity metrics between groups, we conducted permutational multivariate analysis of variance (PERMANOVA) using the vegan package in R. The effect size of variables explaining Bray-Curtis distance was quantified using ADONIS with 999 permutations in the vegan package. Heatmaps and cluster analyzes were generated using all OTU values or core abundant OTU values in the pheatmap package (version 2.30.0) from Bioconductor and the vegan package in R. Average linkage hierarchical clustering and Bray-Curtis distance were utilized for cluster analysis and heatmap generation, respectively.^52^ Differentially abundant taxa were identified using the DESeq2 package (version 1.32.0),^51^ based on the negative binomial (Gamma – Poisson) distribution. For this analysis, the raw microbiome abundance data were transformed into DESeq2 dds objects using the deseq package in R (version 1.32.0). Contrasts were established based on outcome groups, specifically Severe Symptoms (SS) versus No Symptoms (NS). DESeq2 identifies differentially expressed OTUs by measuring the ‘effect size’, represented as the Log2 fold change (LFC), indicating how bacterial abundance differs between SS and NS groups or vice versa. To determine the statistical significance of differentially abundant bacteria, the associated p-value was calculated using the Wald test. The Wald statistic value is obtained by dividing the LFC by its standard error, and this statistic is used to calculate the p-value. To mitigate false positives due to multiple testing, DESeq2 employs the Benjamini and Hochberg/FDR method by default. Differentially abundant species with a false discovery rate (FDR) of 0.05 or less are considered significant.

### GMB database preparation and bacterial identification

The GMB database was constructed from a species-level identified 16S rRNA sequence library of GMB (Gut Microbe Bank), which contains 1,779 microbial species sourced from human gut microbiomes (https://www.gmbank.org). To create this database, we utilized the BLAST(+) command-line tool provided by NCBI.^52^ All reference species sequences were compiled into a fasta file, and the blast db application was employed to generate the GMB Database. Subsequently, differentially abundant OTU sequences obtained from raw data were extracted and used as query sequences against the GMB Database.

### Co-occurrence network construction

To investigate how COVID-19 infection influences microbial co-occurrence relationships, we utilized CoNet,^50^ a Java Cytoscape plug-in, to construct permutation-renormalization-bootstrap networks. Three distinct networks were generated by partitioning the OTU abundance matrix into SS, MS, and NS groups, representing severe symptom, mild symptom, and no symptom groups, respectively. Microbial networks and their connections or edges were derived from OTU occurrence data. CoNet employed multiple ensemble correlation methods to identify significant co-presences across the samples. Five similarity measures, including Spearman and Pearson correlation coefficients, Mutual Information Score, Bray-Curtis, and Kullback-Leibler Dissimilarity, were computed by CoNet to create an ensemble network without a p-value merge. The corrected p-values were obtained using the Benjamini – Hochberg correction method (adjusted *p*-value < .05). Relationships between two OTUs were considered significant in the final network if at least two of the five metrics suggested significant co-abundance. The final co-occurrence network model was visualized using the igraph package in R. The Louvain algorithm was implemented to identify communities within each network, maximizing the modularity score of each OTU within a given network.^51^

### Phylogenetic tree construction

The 16S rRNA gene sequences were aligned with similar sequences from the NCBI nucleotide database.^53^ A phylogenetic tree was then constructed using the maximum likelihood method with 500 bootstrap replicates in the MEGA 11 (Molecular Evolutionary Genetics Analysis) program.^54^

### Transplantation of the microbial cultures and colonization

*Oribacterium* sp. GMB0313 and *Ruminococcus* sp. GMB0270 were cultured according to the supplier’s instructions using appropriate media under anaerobic conditions (GMB, South Korea). The cultures were washed and resuspended in phosphate-buffered saline (PBS) oral administration to hamsters. The prepared microorganisms were orally administered to hamsters at a concentration of 1 × 10^9^ CFU of microorganisms in 100 μL of PBS per day for two weeks.^55^ General health was assessed, including any signs of illness, changes in body weight, fluid consumption, and diarrhea. Stool samples for quantitative bacteriological analysis were collected on Day 0 and Day 2 after the last dose of the study drug. A total of 100 mg of feces was suspended in 1 mL of PBS and incubated at 37 ± 2°C on agar plates for 24–72 hours. After Gram strain testing, individual colonies were analyzed by PCR and sequencing.^56^ PCR was performed at 94°C for 10 min, followed by 40 cycles at 94°C for 60 sec, 50°C for 60 sec, and 72°C for 90 sec.

### Cytokine analysis

Whole blood was collected via cardiac puncture into a 1.5 mL Eppendorf tube. The blood was then centrifuged at 2,500 × g for 10 minutes to separate the serum, which was stored at 25°C until further use. Cytokine concentrations in the serum samples were determined using ELISA kits (Hamster ELISA kit, MyBioSource) following previously established protocols. In brief, the sera were diluted tenfold with dilution buffer and used to quantitate cytokines in a micro-ELISA strip plate pre-coated with mouse anti-cytokine monoclonal antibody. Cytokine concentrations were calculated from the optical density (OD) values of each sample using a standard curve.^44^

### Flow cytometric analysis

Peripheral blood mononuclear cells (PBMCs) were isolated by density gradient centrifugation and stored in liquid nitrogen until further use. Cells were analyzed by staining for intracellular cytokines and cell surface markers. PBMCs were stained with Alexa Fluor 700 anti-hamster CD3e (Thermo Fisher Lot #56003382), APC anti-hamster CD4 (Thermo Fisher Lot #17004182), FITC anti-hamster CD8a (Thermo Fisher Lot #1 1,008,182), PE-Cy7 anti-hamster CD14 (Thermo Fisher Lot # 25014182), and PE anti-hamster CD16 (Thermo Fisher Lot # MHCD1604). Performed at 4°C for 20 minutes.^58,^ Flow cytometry was performed on a high-end Performance Flow cytometry System FACS Symphony A3 (Becton Dickinson, New Jersey, USA) instrument and assessed using Flow Jo v. 10.8.1 software.^57^

### Statistical analysis

The statistical significance of differences between SARS-CoV-2-infected and mock-treated samples was assessed using one-way analysis of variance (ANOVA) followed by multiple comparison testing. Statistical analyses were conducted using GraphPad Prism 8 software (GraphPad Software, La Jolla, CA, USA). A *p*-value less than 0.05 was considered statistically significant.

## Supplementary Material

GM Supplymentary material.docx

## Data Availability

Sequence data generated in this study was uploaded to the NCBI SRA database. It can be accessed via the accession number PRJNA1011488. The data that support the findings of this study are available on request from the corresponding author, Seong-Tshool Hong, upon reasonable request.
