# Supplementary material for: The gut microbe pair of Oribacterium sp. GMB0313 and Ruminococcus sp. GMB0270 confers complete protection against SARS-CoV-2 infection by activating CD8+ T cell-mediated immunity
Source: Gut Microbes. 2024 Apr 18;16(1):2342497. doi: 10.1080/19490976.2024.2342497 (PMC11028030; doi:10.1080/19490976.2024.2342497)
Supplement: GM Supplymentary material.docx [file KGMI_A_2342497_SM4130.docx]

The gut microbe pair of *Oribacterium* sp*.* GMB0313 and *Ruminococcus* sp*.* GMB0270 confers complete protection against SARS-CoV-2 infection by activating CD8+ T cell-mediated immunity

**
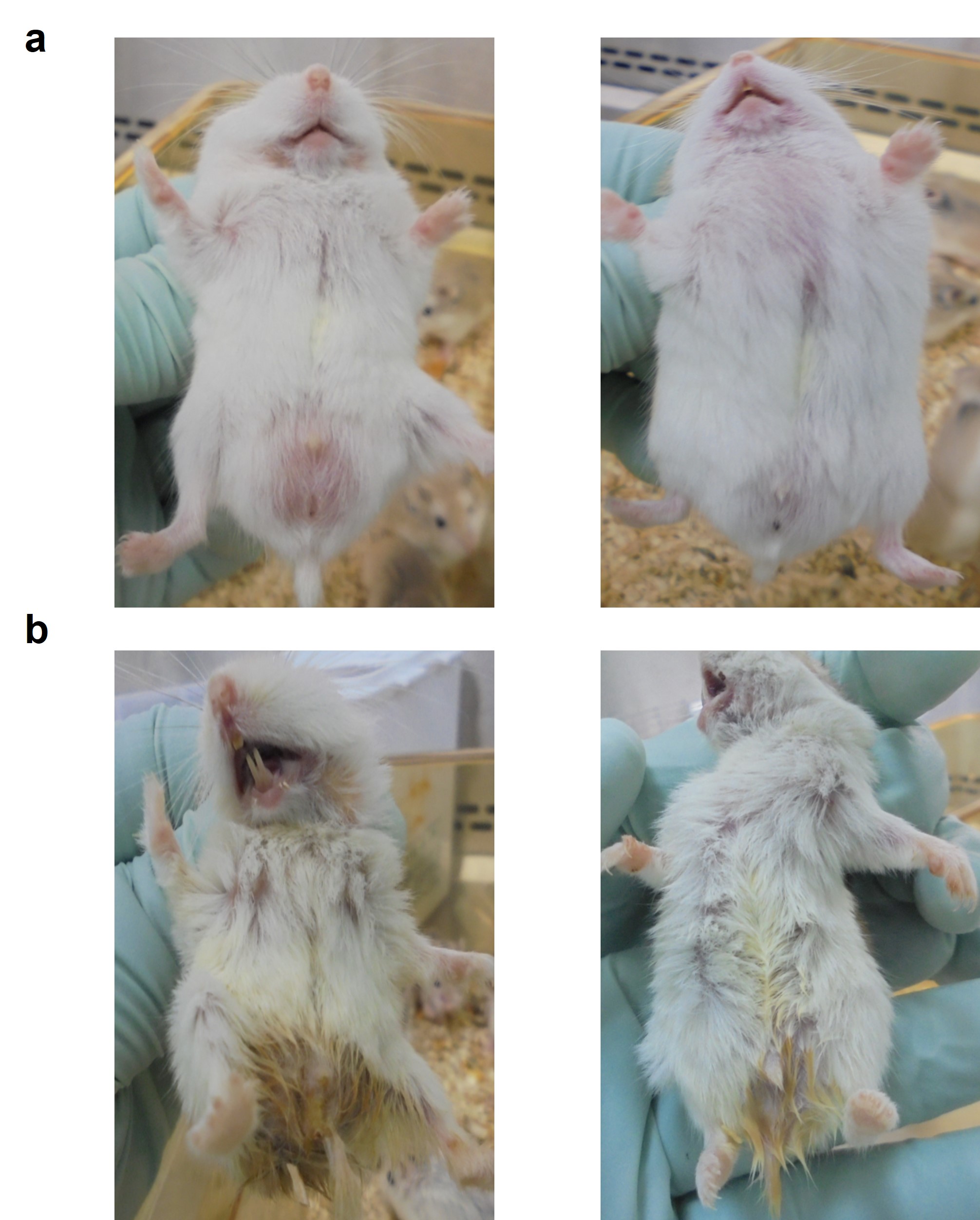
Supplementary Figures and Tables**

**Supplemental Figure S1**. Hamsters infected with SARS-CoV-2 are prone to diarrhea.

(a) Healthy hamsters with normal abdominal hair and no symptoms of diarrhea. (b) SARS-CoV-2-infected hamsters experiencing severe diarrhea prior to death.


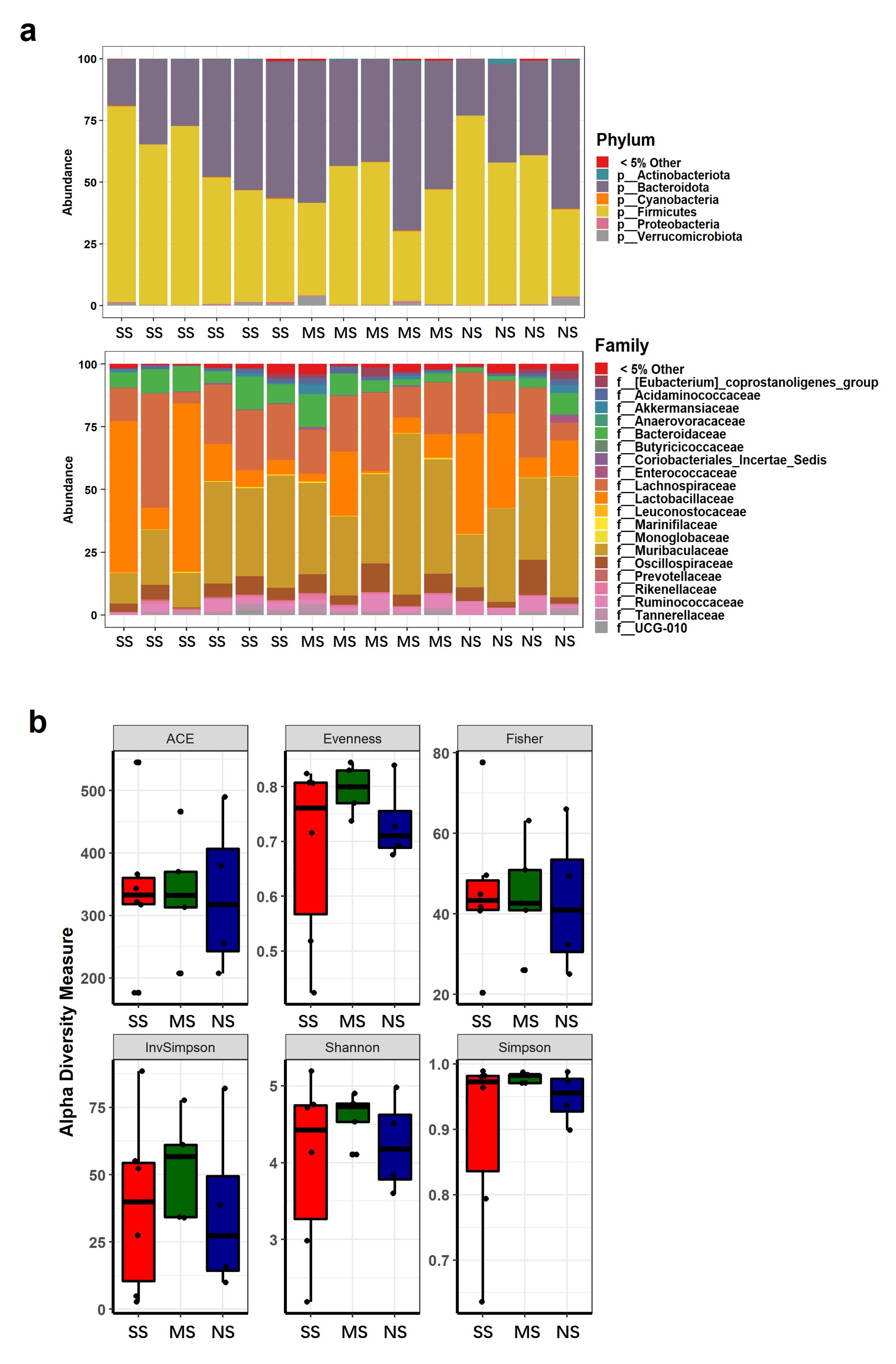


**Supplemental Figure S2.** Heterogeneous distribution of the gut microbiota among the infected SH101 hamsters. (a) Relative abundance of bacterial communities at the phylum and order level for each hamster (n = 15) identified by 16S rRNA gene amplicon sequencing. (b**)** Alpha diversity of each group is presented as ACE, Evenness, Fisher, InvSimpson, Shannon, and Simpson. SS, MS, and NS represent the Severe Symptom, Mild Symptom, and No Symptom groups after challenge with SARS-CoV-2.

**
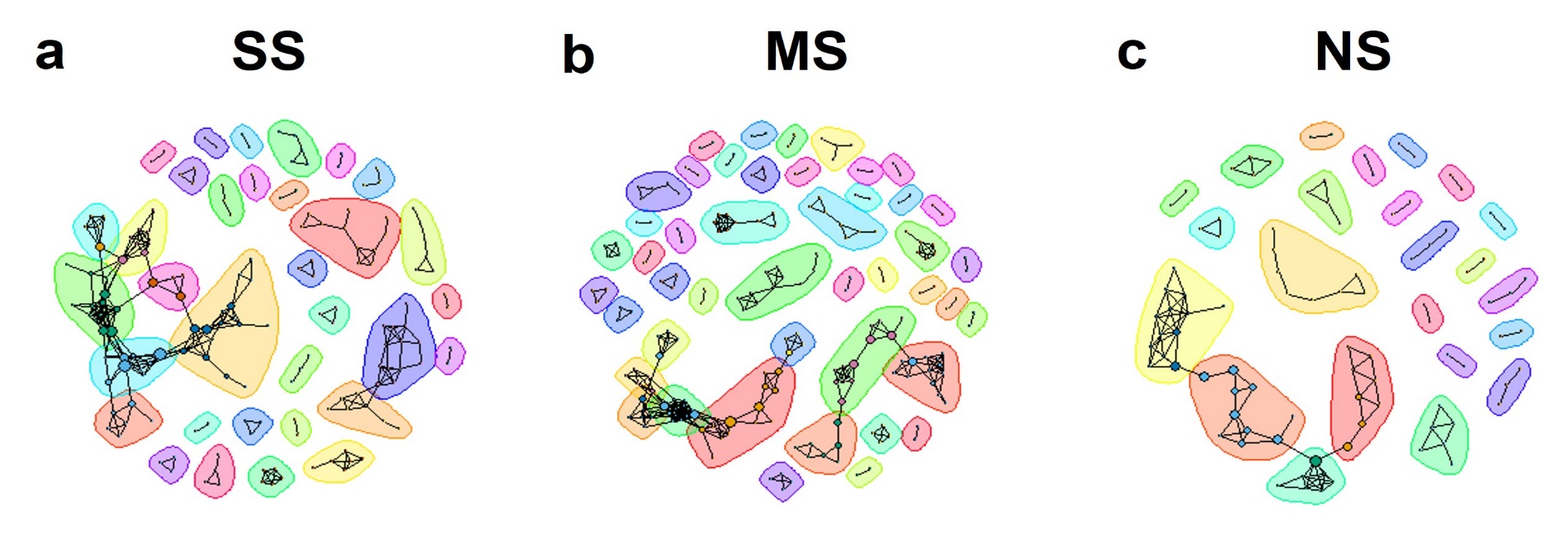
**

**Supplemental Figure S3**. Co-occurrence network analysis was conducted using the ReBoot algorithm for the (a) SS, (b) MS, and (c) NS groups. Color-coded network graphs represent the co-occurrence interactions among OTUs. Transparent shapes represent network communities determined by the Louvain modularity algorithm. The total numbers of OTUs consisting of the gut microbiome of each experimental group were n=974, n=869, and n=802 in SS, MS, and NS, respectively. SS, MS, and NS represent the Severe Symptom, Mild Symptom, and No Symptom groups after challenge with SARS-CoV-2.

**
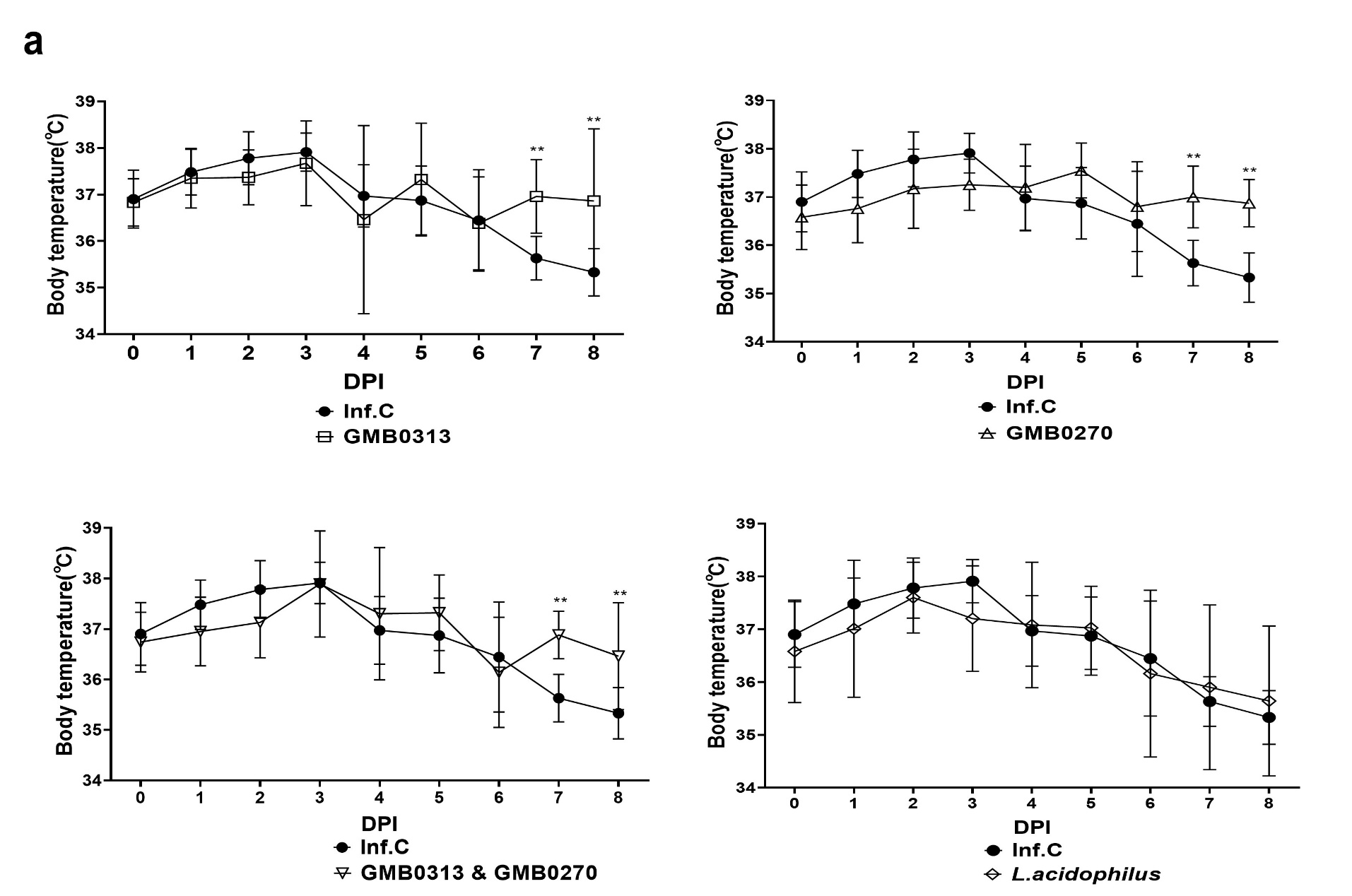
**


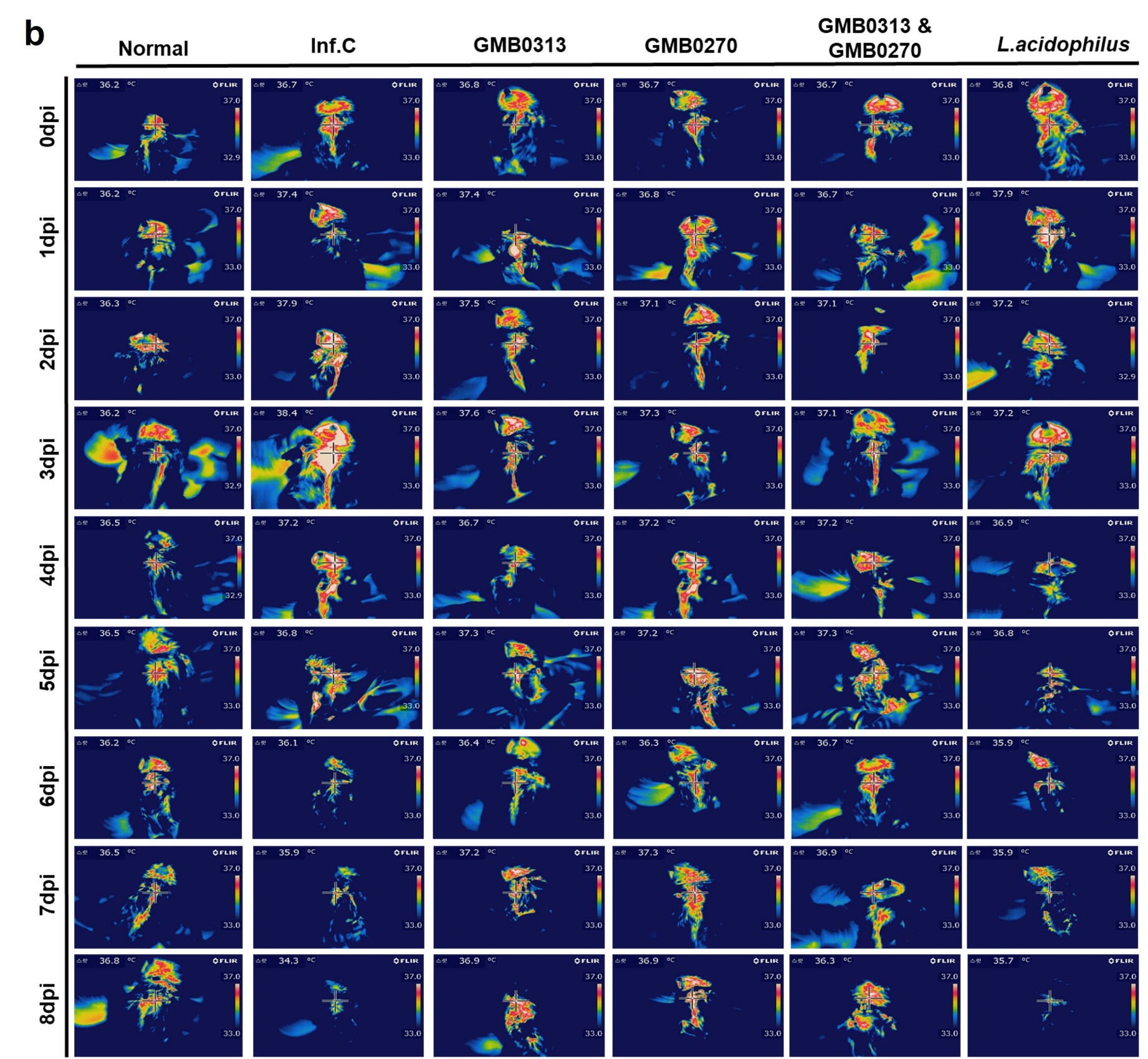


**Supplemental Figure S4**. Body temperature changes and heat sensing images of hamsters infected with SARS-CoV-2. (a) Body temperature changes. (b) Representative images using an infrared thermal induction camera with hamsters fed gut microbe *Oribacterium* sp*.* GMB0313, *Ruminococcus* sp*.* GMB0270, the pair of *Oribacterium* sp*.* GMB0313 and *Ruminococcus* sp*.* GMB0270, and intestine bacteria *L. acidophilus* days post-infection (dpi) of SARS-CoV-2. Data on graphs are mean ± SD. Significance was statistically analyzed and marked on the graphs as **P* < 0.05 and ***P* < 0.01.

|  | **SS** | **MS** | **NS** |
| --- | --- | --- | --- |
| **Global density** | 0.023 | 0.019 | 0.028 |
| **Assortativity** | - 0.0096 | - 0.0092 | - 0.016 |
| **Modularity** | 0.829 | 0.825 | 0.844 |
| **Community** | 33 | 45 | 23 |
| **Node** | 164 | 190 | 94 |
| **Edge** | 306 | 346 | 125 |

**Supplemental Table 1.** Co-occurrence network characteristics of SS, MS, and NS groups. The analysis of microbial co-occurrence relationships involved using CoNet, a Java Cytoscape plug-in, to create permutation-renormalization-bootstrap networks. Three networks were independently constructed for the SS, MS, and NS groups by splitting the OTU abundance matrix. Multiple ensemble correlation methods in CoNet were employed to identify significant co-presences across the samples, utilizing Spearman and Pearson correlation coefficients, the Mutual Information Score, Bray-Curtis, and Kullback-Leibler Dissimilarity. The p-value was corrected using the Benjamini–Hochberg method (adjusted p-value < 0.05). If at least two of the five metrics suggested significant co-abundance between two OTUs, the relationship was retained in the final network as an edge. The final co-occurrence network model was visualized using the igraph package in R, implementing the Louvain algorithm to identify communities within each network and maximize the modularity score of each OTU within a given network. SS, MS, and NS represent the Severe Symptom, Mild Symptom, and No Symptom groups after challenge with SARS-CoV-2.

|  | **OTU** | **Base**  **Mean** | **log2-Fold Change** | **lfcSE** | **stat** | ***p*value** | ***P* adj** | **GMB**  **number** | **GMB**  **Identification** |
| --- | --- | --- | --- | --- | --- | --- | --- | --- | --- |
| 1 | eOTU0368 | 34.82 | -36.79 | 3.98 | -9.25 | 2.26E-20 | 2.52E-17 | GMB0270 | *Ruminococcus* sp*.* |
| 2 | eOTU0403 | 17.83 | -32.32 | 3.99 | -8.11 | 5.13E-16 | 2.55E-13 | GMB0349 | *Porphyromonas*  *catoniae* |
| 3 | eOTU0298 | 29.1 | -28.05 | 3.98 | -7.05 | 1.77E-12 | 3.78E-10 | GMB0245 | *Hungatella*  *hathewayi* |
| 4 | eOTU0347 | 48.51 | -22.60 | 3.98 | -5.68 | 1.35E-08 | 1.68E-06 | GMB0317 | *Stomatobaculum longum* |
| 5 | eOTU0357 | 20.35 | -21.88 | 3.98 | -5.50 | 3.82E-08 | 4.08E-06 | GMB0349 | *Porphyromonas*  *catoniae* |
| 6 | eOTU0195 | 84.04 | -21.76 | 3.98 | -5.47 | 4.55E-08 | 4.54E-06 | GMB0313 | *Oribacterium* sp*.* |
| 7 | eOTU0393 | 30.55 | -21.61 | 3.98 | -5.43 | 5.64E-08 | 5.27E-06 | GMB0313 | *Oribacterium* sp*.* |
| 8 | eOTU0312 | 69.31 | -19.82 | 3.98 | -4.97 | 6.58E-07 | 5.17E-05 | GMB0245 | *Hungatella*  *hathewayi* |
| 9 | eOTU0285 | 82.75 | -18.56 | 4.00 | -4.64 | 3.46E-06 | 0.000246 | GMB0349 | *Porphyromonas*  *catoniae* |
| 10 | eOTU0574 | 22.86 | -17.95 | 4.01 | -4.47 | 7.74E-06 | 0.000526 | GMB0349 | *Porphyromonas*  *catoniae* |
| 11 | eOTU0591 | 22.05 | -17.51 | 4.02 | -4.36 | 1.32E-05 | 0.000788 | GMB0245 | *Hungatella*  *hathewayi* |
| 12 | eOTU0111 | 317.14 | -15.64 | 4.09 | -3.83 | 0.000130 | 0.006493 | GMB0473 | *Campylobacter*  *gracilis* |

**Supplemental Table 2.** The Deseq2 analysis and GMB Database identification result for the identification of 12 abundant OTUs in NS groups relative to the SS and the MS groups. Differentially abundant taxa were identified using the DESeq2 package (v.1.32.0), which employs the negative binomial distribution. The raw microbiome abundance data were converted to DESeq2 dds objects using the deseq package in R (v.1.32.0). Contrasts were based on outcome groups, comparing NS versus SS. DESeq2 reveals differentially expressed OTUs by their effect size, represented as the Log2 fold change (LFC). The associated p-value for a differentially abundant bacterium was calculated using the Wald test. This test involves dividing the LFC by its standard error, and the resulting Wald statistic is used to compute the p-value. To mitigate false positives, DESeq2 employs the Benjamini and Hochberg/FDR method by default. Differentially abundant species with an FDR of 0.05 or less are considered significant. The baseMean indicates the average of the normalized values, log2FoldChange represents the effect size, and lfcSE denotes the standard error of the log2FoldChange estimate. The Wald test results include stat, pvalue, and padj, representing the Wald statistic, Wald test p-value, and Benjamini-Hochberg/FDR adjusted p-value, respectively. The GMB number was obtained by retrieving the raw sequences of 12 enriched OTUs in the NS group against the GMB Database. GMB identification indicates the identified species based on the MGS sequence against the microbes in the Gut Microbe Bank (GMB) (<https://www.gmbank.org)>. SS, MS, and NS represent the Severe Symptom, Mild Symptom, and No Symptom groups after challenge with SARS-CoV-2.

|  | **OTU** | **Base**  **Mean** | **log2-Fold Change** | **lfcSE** | **stat** | ***p*-value** | ***P* adj** | **GMB**  **number** | **GMB**  **identification** |
| --- | --- | --- | --- | --- | --- | --- | --- | --- | --- |
| 1 | eOTU0577 | 22.24 | 16.47 | 4.06 | 4.06 | 4.99E-05 | 0.002569 | GMB0335 | *Selenomonas*  *artemidis* |
| 2 | eOTU0406 | 46.02 | 16.51 | 4.05 | 4.07 | 4.63E-05 | 0.002469 | GMB0765 | *Bacteroides kribbi* |
| 3 | eOTU0421 | 42.15 | 16.72 | 4.06 | 4.12 | 3.77E-05 | 0.002088 | GMB0349 | *Porphyromonas*  *catoniae* |
| 4 | eOTU0332 | 62.37 | 17.02 | 4.06 | 4.19 | 2.75E-05 | 0.001581 | GMB0402 | *Prevotella micans* |
| 5 | eOTU0506 | 28.71 | 17.69 | 4.05 | 4.37 | 1.23E-05 | 0.000765 | GMB0397 | *Prevotella copri* |
| 6 | eOTU0168 | 183.69 | 17.91 | 4.02 | 4.45 | 8.51E-06 | 0.000553 | GMB0473 | *Campylobacter*  *gracilis* |
| 7 | eOTU0563 | 23.37 | 18.79 | 4.02 | 4.67 | 2.96E-06 | 0.000221 | GMB0317 | *Stomatobaculum*  *longum* |
| 8 | eOTU0527 | 27.71 | 20.01 | 4.01 | 4.99 | 6.12E-07 | 5.08E-05 | GMB0532 | *Uncultured* |
| 9 | eOTU0390 | 42.92 | 20.77 | 3.64 | 5.70 | 1.19E-08 | 1.62E-06 | GMB0355 | *Parabacteroides*  *distasonis* |
| 10 | eOTU0610 | 7.90 | 21.24 | 4.01 | 5.30 | 1.17E-07 | 1.03E-05 | GMB0319 | *Roseburia*  *intestinalis* |
| 11 | eOTU0343 | 53.52 | 22.23 | 4.01 | 5.55 | 2.84E-08 | 3.27E-06 | GMB0385 | *Bacteroides*  *intestinalis* |
| 12 | eOTU0335 | 36.18 | 23.59 | 4.00 | 5.89 | 3.81E-09 | 5.70E-07 | GMB0765 | *Bacteroides kribbi* |
| 13 | eOTU0231 | 49.32 | 24.62 | 4.00 | 6.15 | 7.85E-10 | 1.30E-07 | GMB0349 | *Porphyromonas catoniae* |
| 14 | eOTU0704 | 17.77 | 26.26 | 4.00 | 6.56 | 5.46E-11 | 1.02E-08 | GMB0317 | *Stomatobaculum longum* |
| 15 | eOTU0496 | 9.29 | 32.38 | 4.01 | 8.07 | 7.10E-16 | 2.65E-13 | GMB0245 | *Hungatella hathewayi* |
| 16 | eOTU0456 | 20.32 | 36.87 | 4.00 | 9.21 | 3.37E-20 | 2.52E-17 | GMB0765 | *Bacteroides kribbi* |
| 17 | eOTU0279 | 34.74 | 28.59 | 4.00 | 7.14 | 9.25E-13 | 2.30E-10 | GMB0245 | *Hungatella hathewayi* |
| 18 | eOTU0240 | 45.37 | 8.94 | 2.37 | 3.77 | 0.000161 | 0.007757 | GMB0302 | *Blautia producta* |
| 19 | eOTU0490 | 18.45 | 30.89 | 4.01 | 7.71 | 1.25E-14 | 3.74E-12 | GMB0306 | *Blautia hydrogenotrophica* |

**Supplemental Table 3.** The Deseq2 analysis and GMB Database identification result for the identification of 19 abundant OTUs in SS groups relative to the NS and the MS groups. Differentially abundant taxa were identified using the DESeq2 package (v.1.32.0), based on the negative binomial distribution. Raw microbiome abundance data were converted to DESeq2 dds objects using the deseq package in R (v.1.32.0). Contrasts were based on outcome groups; SS versus NS. The baseMean represents the average of the normalized values, log2FoldChange represents the effect size, and lfcSE is the standard error of the log2FoldChange estimate. The Wald test results are shown as stat, pvalue, and padj, representing the Wald statistic, Wald test p-value, and Benjamini-Hochberg/FDR adjusted p-value, respectively. GMB numbers were obtained by retrieving the raw sequences of 19 enriched OTUs in the SS group against the GMB Database. GMB identification indicates the identified species based on the MGS sequence against the microbes in the Gut Microbe Bank (GMB) (<https://www.gmbank.org>). SS, MS, and NS represent the Severe Symptom, Mild Symptom, and No Symptom groups after challenge with SARS-CoV-2.
